# Analgesia, Sedation, and Neuromuscular Blockade in Pediatric Severe Traumatic Brain Injury: Secondary Analysis of the “Approaches and Decisions in Acute Pediatric TBI Trial” (ADAPT)

**DOI:** 10.1007/s12028-025-02336-8

**Published:** 2025-08-15

**Authors:** Jennifer Clancy Laws, Jaskaran Rakkar, Sandra D. W. Buttram, Michael Seth Wolf

**Affiliations:** 1 Division of Critical Care Medicine, Department of Pediatrics, Vanderbilt University Medical Center, Nashville, TN USA; 2 Division of Critical Care, Department of Child Health, Phoenix Children’s Hospital, Phoenix, AZ USA

**Keywords:** Brain injuries (traumatic), Neuromuscular blockade, Hypnotics and sedatives, Analgesia, Intracranial pressure

## Abstract

**Background:**

Sedative, analgesia, and neuromuscular blocking (NMB) medications may be necessary in the acute management of pediatric severe traumatic brain injury (sTBI), yet limited data exist to guide their use. We aimed to describe the use of continuous infusions of these medications in children with sTBI, to determine temporal trends during the first week of management, and to evaluate associations with in-hospital mortality.

**Methods:**

We conducted a secondary analysis of the Approaches and Decisions in Acute Pediatric Traumatic Brain Injury Trial (NCT04077411, 2014–2017), a prospective multicenter observational study of patients < 18 years old with sTBI (Glasgow Coma Scale ≤ 8) who underwent intracranial pressure monitoring. Continuous analgesic, sedative, and NMB medication infusions administered in the first 7 days after sTBI were analyzed.

**Results:**

Data from 929 patients were analyzed with a median Glasgow Coma Scale of 6 (interquartile range 3–7), 14% hospital mortality. In the 7 days after intracranial pressure monitor placement, 866 (93%) patients received an opioid infusion, with 659 (71%) patients having received fentanyl. A total of 679 (73%) patients received benzodiazepine: 671 (72%) patients received midazolam. A total of 362 (39%) patients received NMB, with the most common being vecuronium, administered to 141 (15%) patients. Propofol was administered to 264 (28%) patients, alpha-2 agonist to 263 (28%) patients, and ketamine to 4 (0.43%) patients. The median number of infusions per patient was 2 (interquartile range 1–2), with the highest number on intensive care unit day 2. Morphine and dexmedetomidine infusions were used more often in survivors than nonsurvivors (33 vs. 16%, and 30 vs. 9%, respectively, *p* < 0.001).

**Conclusions:**

Fentanyl and midazolam were the most common analgesic and sedative continuous infusions during acute pediatric sTBI management. Propofol and dexmedetomidine were used less frequently. Opioid (specifically morphine) and dexmedetomidine infusions were associated with survival. Larger studies are needed to determine the safest and most effective analgesia, sedation, and NMB medication strategy for children with sTBI.

**Supplementary Information:**

The online version contains supplementary material available at 10.1007/s12028-025-02336-8.

## Introduction

Traumatic brain injury (TBI) is a leading cause of childhood morbidity and mortality, accounting for ore than 2,700 childhood deaths per year in the United States [1–5]. Children with severe TBI (sTBI) are at risk of further neuronal death due to expansion of the primary injury and secondary insults such as hypoxia, ischemia, and edema leading to elevated intracranial pressure (ICP) [6]. Given the lack of targeted neuroprotective therapy, treatment is largely supportive, emphasizing prevention and treatment of elevated ICP in accordance with evidence-based guidelines. Analgesic, sedative, and neuromuscular blockade (NMB) medications are commonly administered during sTBI management to facilitate comfort and synchrony with invasive mechanical ventilation, treat pain and anxiety during invasive procedures, and minimize cellular metabolic demand in critical illness and in targeted efforts to treat intracranial hypertension [7].

There is currently a paucity of data to guide appropriate sedative and NMB use in children with sTBI [7, 8]. There is a level III recommendation to avoid bolus doses of midazolam and/or fentanyl during ICP crisis due to risk of cerebral hypoperfusion [9, 10] and to avoid prolonged continuous propofol infusions [8]. In adults with sTBI, propofol is recommended for ICP control and dexmedetomidine is associated with favorable outcome [11, 12]. Given the absence of data, decisions regarding the choice and dosing of analgesic, sedative, and NMB agents are left to the treating physician. This exploratory post hoc secondary analysis of the Approaches and Decisions in Acute Pediatric Traumatic Brain Injury Trial (ADAPT) aims to describe the use of and temporal trends in continuous infusions of analgesic, sedative, and NMB medications during the first 7 days of acute management of children with sTBI and determine the associations of specific infusions with hospital mortality.

## Methods

Data were obtained from the US Federal Interagency Traumatic Brain Injury Research Informatics System repository in this nonprespecified secondary analysis. The institutional review boards of Vanderbilt University Medical Center and Phoenix Children’s Hospital determined our investigation as nonhuman study participants research not requiring informed consent. All data obtained from US Federal Interagency Traumatic Brain Injury Research were deidentified, and all research procedures followed the ethical standards of the Helsinki Declaration of 1975.

### ADAPT Dataset and Analysis

The ADAPT dataset (NCT04077411) is derived from an international prospective multicenter observational cohort study of pediatric patients (aged 0–18 years) with sTBI (Glasgow Coma Scale [GCS] score 3–8) that had ICP monitors placed as the standard of care. Patients who were pregnant or had their ICP monitor placed at a nonrecruiting site were excluded. Each site treated patients based on local institutional guidelines, with treatment data collected over the first 7 days of acute management after ICP monitor placement. Patients were enrolled from February 2014 to September 2017 from 49 international centers. In this secondary analysis, patients were included if they received at least one continuous infusion of analgesic, sedative, and/or NMB medication during the first 7 days after ICP monitor placement. A daily record of continuous infusions was collected for each patient (collection form listed in Supplementary Materials). The class (e.g., opioid, benzodiazepine) and specific medication (e.g., fentanyl, midazolam) of each continuous infusion was analyzed. The number of patients receiving each continuous medication class were then separated by day of treatment to evaluate their use over time and further stratified by specific medication. Hospital mortality or survival to discharge was recorded for each patient. Age, sex, cause and mechanism of injury, presence of abusive head trauma (AHT) [13], admission GCS score, abbreviated injury score, Pediatric Risk of Mortality (PRISM) III score, and frequency of exposure to each medication infusion class (including opioids, benzodiazepines, NMB medications, propofol, alpha-2 agonists, and other) and specific medication infusion (including fentanyl, morphine, remifentanil, hydromorphone, midazolam, lorazepam, vecuronium, rocuronium, cisatracurium, atracurium, propofol, dexmedetomidine, clonidine, and ketamine) were compared between survivors and nonsurvivors. Barbiturates were analyzed separately given their use as a second-line therapy for refractory intracranial hypertension [8].

### Statistical Analysis

Demographic, injury characteristics, clinical data, and outcomes were expressed as frequencies and percentages for categorical variables and as medians and interquartile ranges for continuous variables. Continuous data were evaluated using Shapiro–Wilk’s method to assess normality. Differences between survivors and nonsurvivors were evaluated using Pearson’s *χ*^2^ test for categorical data and unpaired Wilcoxon rank-sum test for continuous variables. In this nonprespecified hypothesis generating retrospective analysis, statistical significance was defined a priori as a *p* value less than 0.05. For each medication found to have association with hospital mortality, a multivariable logistic regression model was created to test for independence of association. Variables included in the models were age; admission GCS, abbreviated injury score, and PRISM III scores; median pediatric intensity level of therapy (PILOT) score; status epilepticus diagnosis; barbiturate use; and intensive care unit (ICU) length of stay, with center included as a random effect to account for variability between sites. Given that AHT is associated with younger age, longer length of stay, and greater odds of death and new impairment [14], AHT was excluded from the model. To assess potential collinearity, we calculated pairwise Pearson correlation coefficients among all continuous and ordinal model variables and excluded those with high correlation. All statistical analyses were performed using RStudio version 2024.04.1 + 748 (RStudio, Boston, MA) and R version 4.3.3 (R Foundation for Statistical Computing, Vienna, Austria) with the following packages: tidyverse, lubridate, lme4, gtsummary, flextable, officer, and patchwork.

## Results

A total of 929 patients from the ADAPT dataset met criteria for inclusion in this secondary analysis. In the first 7 days after ICP monitor placement, 866 (93%) patients received a continuous opioid infusion, and 679 (73%) received a continuous benzodiazepine infusion. Less common sedative infusions included 264 (28%) patients receiving propofol 263 (28%) receiving alpha-2 agonists, and 4 (0.43%) receiving ketamine, respectively. Overall, 362 (39%) patients received continuous NMB infusions. The median number of infusions was 2 (interquartile range 1–2) during the study period. Table 1 summarizes demographic and clinical characteristics stratified by hospital mortality. Cause of injury was significantly different between survivors and nonsurvivors, and suspected AHT was more common in nonsurvivors. Survivors also had higher admission GCS scores, lower PRISM III scores, and longer hospital and ICU lengths of stay than nonsurvivors.

**Table 1:** Study population stratified by mortality

Characteristic	Overall (*N* = 929)	Survived (*n* = 798)	Died (*n* = 131)	*p* value^a^
Age, median (IQR) (yr)	7.0 (3.0–12.0)	7.0 (3.0–12.0)	6.0 (2.0–12.0)	0.351
Sex, *n* (%)				> 0.999
Male	598 (64)	514 (64)	84 (64)	
Female	331 (36)	284 (36)	47 (36)	
Cause, *n* (%)				0.002*
MVA	528 (57)	460 (58)	68 (52)	
Fall	171 (18)	149 (19)	22 (17)	
Abuse	141 (15)	107 (13)	34 (26)	
Other accidental	89 (10)	82 (10)	7 (5)	
Mechanism, *n* (%)				0.673
Impact	593 (64)	516 (65)	77 (59)	
Fall	168 (18)	145 (18)	23 (18)	
Accel/Decel	86 (9)	72 (9)	14 (11)	
Gunshot	47 (5)	37 (5)	10 (8)	
Crush	16 (2)	13 (2)	3 (2)	
Unknown	10 (1)	8 (1)	2 (2)	
Other	9 (1)	7 (1)	2 (2)	
Concerns for AHT, *n* (%)	114 (12)	88 (11)	26 (20)	0.007*
Admission GCS score, median (IQR)	6.0 (3.0–7.0)	6.0 (3.0–7.0)	3.0 (3.0–6.0)	< 0.001*
Abbreviated injury score, median (IQR)	8.0 (5.0–11.0)	8.0 (5.0–11.0)	8.0 (5.0–12.0)	0.298
PRISM III, median (IQR)	12.0 (8.0–20.0)	11.0 (7.0–18.0)	21.0 (15.0–29.0)	< 0.001*
Medications (infusion), *n* (%)				
Opioid	866 (93)	751 (94)	115 (88)	0.013*
Benzodiazepine	679 (73)	590 (74)	89 (68)	0.184
Neuromuscular blockade	362 (39)	310 (39)	52 (40)	0.930
Propofol	264 (28)	234 (29)	30 (23)	0.160
Alpha-2 agonist	263 (28)	251 (31)	12 (9)	< 0.001*
Ketamine	4 (0)	4 (1)	0 (0)	0.927
Number of infusions (per medication class), median (IQR)	2.0 (1.0–2.0)	2.0 (1.0–2.0)	1.0 (1.0–2.0)	< 0.001*
ICU length of stay, median (IQR)	11.0 (6.0–16.0)	11.5 (7.0–17.0)	5.0 (3.0–9.0)	< 0.001*
Hospital length of stay, median (IQR)	19.0 (11.0–31.0)	21.0 (13.0–32.0)	5.0 (3.0–11.0)	< 0.001*
GOS-E Peds, median (IQR)	5.0 (2.0–6.0)	3.0 (2.0–6.0)	8.0 (8.0–8.0)	< 0.001*

^*^p < 0.05

Following the convention by Ferguson et al. [13], we combined study participants with “probable AHT” and “definite AHT” into an AHT group and “possible AHT” study participants into the no AHT group. Accel, acceleration; AHT, abusive head trauma; Decel, deceleration; GCS, Glasgow Coma Scale; GOS-E Peds, pediatric Glasgow Outcome Scale Extended; ICU, intensive care unit; IQR, interquartile range; MVA, motor vehicle accident; PRISM, Pediatric Risk of Mortality

^a^Wilcoxon rank-sum test; Pearson’s *χ*^2^ test

Table 2 summarizes data for each specific medication infusion. Use of each specific medication infusion was not mutually exclusive given that some patients were exposed to multiple agents in each class of medication. Of the 929 total patients, 659 (71%) patients received fentanyl, and 286 (31%) received morphine infusions. Within the class of benzodiazepines, 671 (72%) of all patients received midazolam as an infusion. The frequency of specific NMB infusions was fairly equivalent, with 141 (15%) patients receiving vecuronium, 116 (12%) receiving rocuronium, and 102 (11%) receiving cisatracurium.

**Table 2:** Medication details stratified by mortality

Medication infusion	Overall (*N* = 929)	Survived (*n* = 798)	Died (*n* = 131)	*p* value^a^
Opioid, *n* (%)				
Fentanyl	659 (71)	565 (71)	94 (72)	0.905
Morphine	286 (31)	265 (33)	21 (16)	< 0.001*
Remifentanil	59 (6)	54 (7)	5 (4)	0.276
Hydromorphone	37 (4)	36 (5)	1 (1)	0.073
Benzodiazepine, *n* (%)				
Midazolam	671 (72)	583 (73)	88 (67)	0.198
Lorazepam	20 (2)	19 (2)	1 (1)	0.391
Neuromuscular blockade, *n* (%)				
Vecuronium	141 (15)	121 (15)	20 (15)	> 0.999
Rocuronium	116 (12)	97 (12)	19 (15)	0.541
Cisatracurium	102 (11)	88 (11)	14 (11)	> 0.999
Atracurium	12 (1)	12 (2)	0 (0)	0.320
Propofol, *n* (%)	264 (28)	234 (29)	30 (23)	0.160
Alpha-2 agonist, *n* (%)				
Dexmedetomidine	251 (27)	239 (30)	12 (9)	< 0.001*
Clonidine	13 (1)	13 (2)	0 (0)	0.285
Ketamine, *n* (%)	4 (0)	4 (1)	0 (0)	0.927

^*^p < 0.05

^a^Pearson’s *χ*^2^ test

**Fig. 1:**
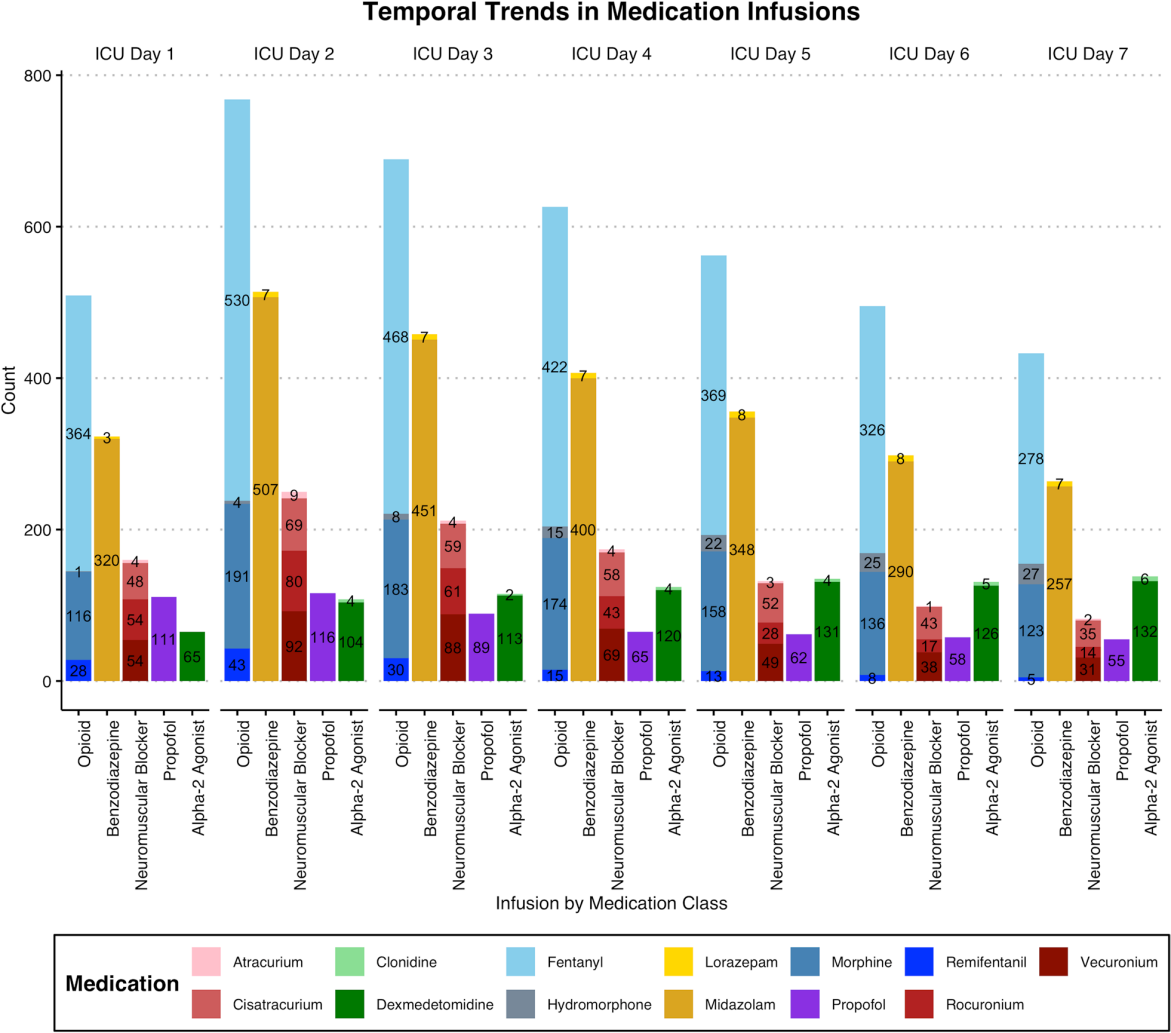
Temporal trends of continuous medication use over the first 7 days of pediatric ICU admission. Continuous infusions are stratified by medication class then further delineated by specific medication subtype. The number of patients receiving each infusion on each day is displayed over the corresponding bar. Because of low numbers, ketamine is omitted from the graph. The number of patients receiving ketamine infusions each day was day 1 = 0 patients, day 2–5 = 1 patient, day 6 = 2 patients, and day 7 = 3 patients, respectively. ICU, intensive care unit

**Fig. 2:**
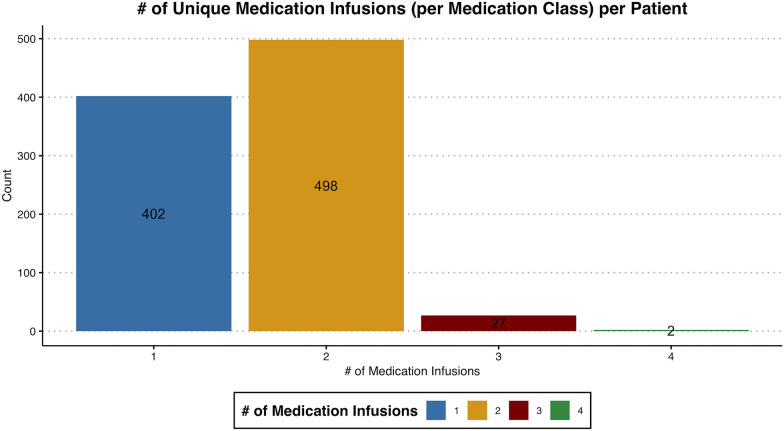
Number of total unique medication infusions (per medication class) per patient

**Fig. 3:**
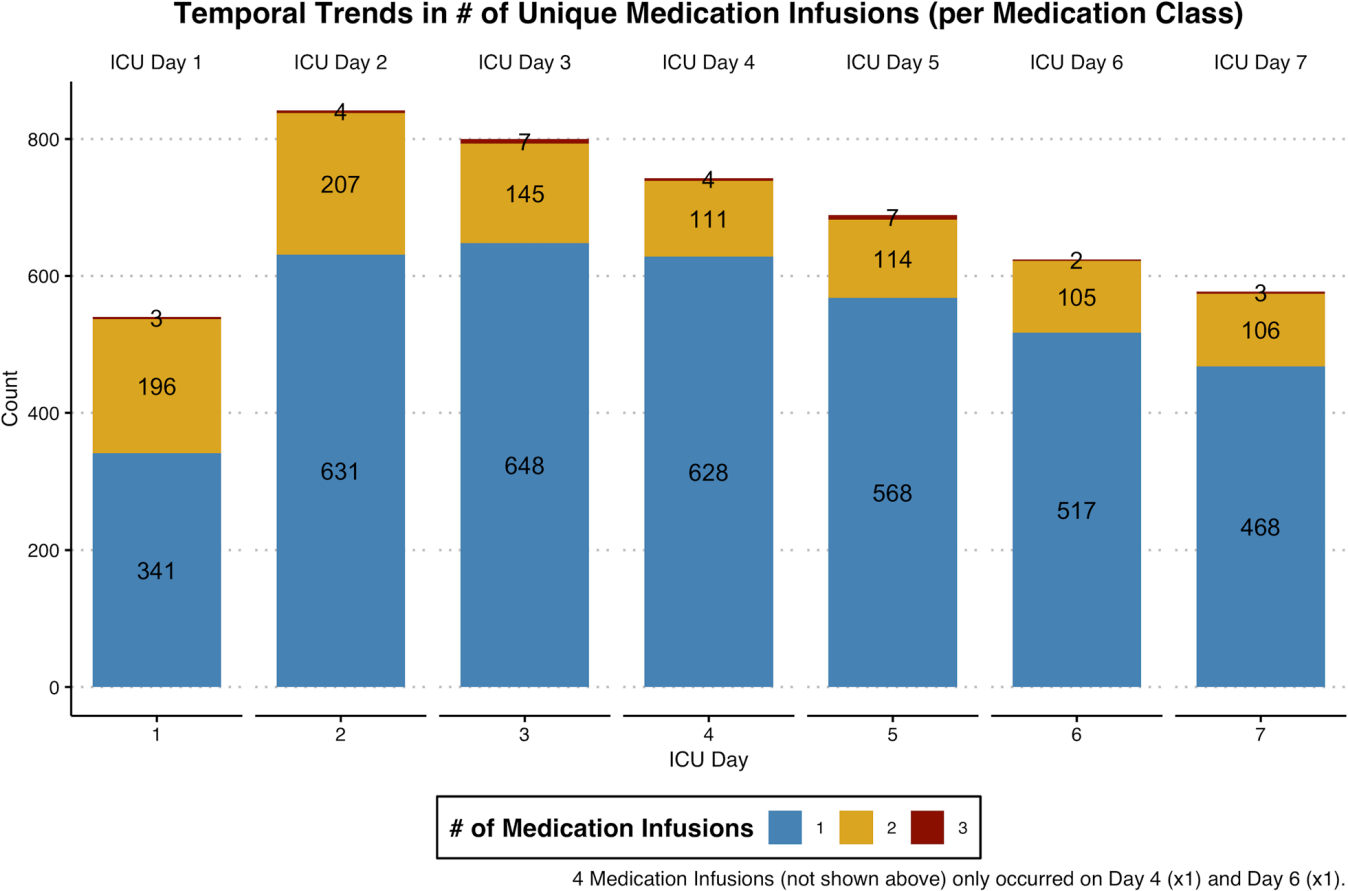
Temporal trend of number of continuous medication infusion per medication class during first 7 days of pediatric ICU admission. ICU, intensive care unit

Temporal trends in medication administration are displayed in Fig. 1. The use of continuous opioid, benzodiazepine, and NMB infusions was highest on day 2, with a general trend of decreased usage on subsequent days. Propofol use was highest on days 1 and 2 and decreased on subsequent days. Alpha-2 agonist agents had a consistent incremental increase in usage over time. A majority of patients received two infusions, followed in frequency by those receiving only one infusion (Fig. 2). The absolute number of infusions was lowest on day 1, with a peak on day 2 and then a subsequent decrease from days 3–7 (Fig. 3).

### Medication Infusions Stratified by Hospital Mortality

Overall, 798 (86%) of patients survived to hospital discharge. Patients who survived were more likely to receive an opioid infusion compared with those who died (94 vs. 88%, *p* = 0.013). Morphine infusions were more common in patients who survived versus those who died (33 vs. 16%, *p* < 0.001), as were dexmedetomidine infusions (30 vs. 9%, *p* < 0.001). There were no differences in benzodiazepine, propofol, or NMB infusion use between patients who survived versus those who died.

Two multivariable logistic regression models to assess morphine and dexmedetomidine infusions for independent association with mortality are shown in Tables 3 and 4, respectively. Both models showed that higher admission PRISM III score and barbiturate use were associated with increased mortality and higher admission GCS score and longer ICU length of stay were associated with decreased mortality. Morphine was associated with 0.38 lower odds of mortality (95% confidence interval 0.20–0.70). Dexmedetomidine was associated with 0.32 lower odds of mortality (95% confidence interval 0.16–0.60). The correlation matrix demonstrated low to moderate correlations among predictor variables, with Pearson coefficients ranging from − 0.29 to + 0.30 (Supplemental Table 1).

**Table 3:** Factors associated with mortality after sTBI: model with morphine infusion

Characteristic	Multivariable logistic regression: mortality in children after sTBI	
	OR (95% CI)	*p* value
Age (yr)	1.00 (0.95–1.04)	0.87
Admission GCS score	0.80 (0.69–0.92)	0.002*
Admission AIS score	1.04 (0.98–1.10)	0.16
Admission PRISM III score	1.14 (1.10–1.17)	< 0.001*
Median PILOT score	1.12 (0.95–1.32)	0.18
Status epilepticus		
No	–	
Yes	1.25 (0.48–3.28)	0.65
Barbiturate use		
No	–	
Yes	3.04 (1.78–5.19)	< 0.001*
ICU LOS (days)	0.88 (0.85–0.91)	< 0.001*
Morphine infusion		
No	–	
Yes	0.38 (0.20–0.70)	0.002*

^*^p < 0.05

AIS*,* Abbreviated Injury Score; CI, Confidence Interval; GCS, Glasgow Coma Scale; ICU, Intensive Care Unit; LOS, Length of Stay; OR, Odds Ratio; PILOT, pediatric intensity level of therapy; PRISM, Pediatric Risk of Mortality; sTBI, Severe Traumatic Brain Injury

**Table 4:** Factors associated with mortality after sTBI: model with dexmedetomidine infusion

Characteristic	Multivariable logistic regression: mortality in children after sTBI	
	OR (95% CI)	*p* value
Age (yr)	1.00 (0.95–1.05)	> 0.99
Admission GCS score	0.81 (0.71–0.92)	0.002*
Admission AIS score	1.02 (0.97–1.08)	0.39
Admission PRISM III score	1.14 (1.11–1.17)	< 0.001*
Median PILOT score	1.11 (0.95–1.30)	0.18
Status epilepticus		
No	–	
Yes	1.64 (0.60–4.03)	0.31
Barbiturate use		
No	–	
Yes	2.46 (1.50–4.05)	< 0.001*
ICU LOS (days)	0.90 (0.86–0.92)	< 0.001*
Dexmedetomidine infusion		
No	–	
Yes	0.32 (0.16 to 0.60)	< 0.001*

^*^p < 0.05

AIS, abbreviated injury score; CI, confidence interval; GCS, Glasgow Coma Scale; ICU, intensive care unit; LOS, length of stay; OR, odds ratio; PILOT, pediatric intensity level of therapy; PRISM, Pediatric Risk of Mortality; sTBI, severe traumatic brain injury

## Discussion

This secondary analysis of the ADAPT dataset describes the use of continuous analgesic, sedative, and NMB medication infusions in a large, international cohort of children with sTBI during their first 7 days of ICU management after ICP monitor placement. The predominant classes of medications in this population were opioids and benzodiazepines, with the most common agents within these classes being fentanyl and midazolam. More than one third of patients received NMB infusions, similar to a previous trial in pediatric sTBI [15]. The number of continuous analgesic, sedative, and NMB infusions per patient peaked on day 2 and incrementally decreased from days 3–7. For patients who required multiple infusions, most patients required two infusions on days 1–3, with subsequent decrease in the need for multiple infusions from days 4–7. Patients who survived were more likely to receive opioid (specifically morphine) and alpha-2 agonist infusions (specifically dexmedetomidine) compared with nonsurvivors.

The absence of high-level recommendations and paucity of data to guide analgesia, sedation, and NMB in pediatric sTBI presents clinicians with a formidable challenge. In this study, we found that fentanyl and midazolam are by far the most common analgesic and sedative infusions and the only two to be given to the majority of pediatric patients with sTBI in the ADAPT dataset. It is conceivable that continuous infusions of these medications have physiologic effects distinct from those of bolus doses. In an animal model of TBI, fentanyl infusions resulted in preserved cerebral blood flow despite hypotension [16]. Morphine infusions can cause peripheral vasodilation and significant hypotension, partly due to plasma histamine release [17]. In contrast, fentanyl infusions do not affect plasma histamine levels and are not associated with a decrease in arterial pressure or systemic vascular resistance. Additionally, some degree of renal impairment is common in pediatric ICU patients with moderate to severe TBI [18, 19]. Prolonged morphine use can lead to redistribution, accumulation, and unpredictable delays in awakening, especially with an increased half-life in the setting of renal injury. In contrast, fentanyl is metabolized to inactive metabolites, leading to less accumulation in renal insufficiency, making it a potentially better drug choice. High-dose midazolam infusions resulted in lower cerebral blood flow in dogs, but this was accompanied by reduced cerebral metabolic rate [20]. Midazolam has a shorter context-sensitive half-life, with rapid onset and offset of action, and may allow for faster awakening and neurological assessment of patients with TBI as compared with other benzodiazepines [21]. Taken together, these observations highlight the need to study the effects of analgesia, sedative, and NMB strategies on ICP and clinical outcomes.

Our study observed that opioid infusions were more common in those who survived versus those who died, with morphine infusions associated with decreased odds of mortality. In animal models, morphine has shown possible neuroprotective effects such as reducing brain edema and neuroinflammatory cytokines [22]. It is possible that morphine was more common in survivors due to opioid rotation after developing tachyphylaxis to an alternative opioid while on a prolonged course of an opioid infusion. However, the temporal trend of morphine follows a similar trend to fentanyl, with usage being highest on day 2 and then gradually decreasing on the following days.

Our analysis reveals that only a minority of pediatric patients in the ADAPT dataset received propofol and/or dexmedetomidine. Propofol is recommended for the treatment of elevated ICP in adults with sTBI, given its rapid onset, short duration of action, and potential neuroprotection by suppression of cerebral metabolism. In a randomized trial comparing morphine with propofol in adults with TBI, daily mean ICP and cerebral perfusion pressure (CPP) were similar between the propofol and morphine groups, and ICP on day 3 was lower for the propofol group [23]. In an observational study, propofol was associated with higher CPP in adults [24]. Caution is advised because high doses or prolonged infusions can cause hypotension and propofol infusion syndrome [12, 25, 26]. Current pediatric TBI guidelines recommend against propofol, citing a US Food and Drug Administration warning [8, 27]. It should be noted that propofol dose information, associated physiologic values, or potential propofol infusion syndrome data were not available for our study. Therefore, although propofol was used in a small subset of patients in this study, we cannot draw conclusions about its safety or physiologic effects in pediatric patients with sTBI.

In recent years, dexmedetomidine has gained favor as a sedative in critically ill adults and children because it offers an agent that provides both analgesia and sedation with more delirium-free and coma-free days [12, 28, 29]. Because of insufficient evidence, neither the adult nor pediatric TBI guidelines mention dexmedetomidine. In a retrospective cohort analysis, dexmedetomidine use was associated with increased hospital and 6-month survival in adult patients with TBI with moderate to sTBI [30]. In a recent retrospective propensity-weighted analysis, Liu et al. [11] observed improved 6-month functional outcomes with early dexmedetomidine exposure in adult patients with TBI requiring ICP monitoring. Of the 251 patients who received dexmedetomidine in our study, 239 (95%) survived, which suggests that dexmedetomidine is safe in the sTBI population. Dexmedetomidine can also be used to treat paroxysmal sympathetic hyperactivity (PSH), which is a common sequela of sTBI. Dexmedetomidine has been reported to reduce frequency and severity of PSH symptoms, although PSH is not typically diagnosed within the first 7 days after TBI [31, 32]. This study is unable to ascertain the specific indications for selection or avoidance of dexmedetomidine, such as bradycardia, and further in-depth analysis is warranted to characterize the effects of dexmedetomidine on physiology and clinical outcome in pediatric patients with sTBI.

Although ketamine has previously been contraindicated due to early reports of increased ICP [33, 34], current pediatric TBI guidelines offer no specific recommendation for or against ketamine [7, 8]. A recent study in the adult TBI population found that ketamine exposure was not associated with worse survival despite being administered to patients with more severe injury [35]. Additionally, ketamine exposure was associated with reduced ICP and lower concentration of TBI biomarkers glial fibrillary acidic protein and microtubule-associated protein 2. Two pediatric studies offer preliminary evidence suggesting an association with lower ICP and higher CPP after ketamine administration in the setting of elevated ICP [36, 37]. A recent retrospective pediatric study of ketamine in refractory status epilepticus included four patients with TBI [38]. We observed that ketamine infusions were rare but not absent in the ADAPT dataset; four patients received ketamine, and all survived to hospital discharge. Future studies are warranted to determine the potential role of ketamine as a sedative for children with sTBI.

This hypothesis generating retrospective observational analysis provides insight into sedation practices in the pediatric sTBI population. However, our study has multiple limitations. First, the ADAPT dataset relies on a daily checklist of analgesic, sedative, and NMB medications (listed in the Supplemental Materials). Dexmedetomidine and ketamine had no dedicated checkbox, and were manually entered as “Sedative, Other,” which may have impacted reporting accuracy. The data entry format did not allow us to discern whether multiple infusions in a single day represented cross-titration versus additive therapy. Second, more granular data concerning the timing, dosage, escalation, de-escalation, and clinical indication were not available. We were unable to evaluate the association between medication infusions and acute physiologic and clinical changes (e.g., ICP, blood pressure, heart rate, level of sedation). Future prospective studies might evaluate the physiologic effects of boluses and continuous infusions of each medication. Third, we were unable to account for vasoactive infusions, hyperosmolar therapy, and other interventions that may have influenced medication selection. Fourth, although we observed differences in the proportion of some medications given to patients who survived versus those who died, it is impossible to infer causality. These differences may be attributed to beneficial medication effects or to confounding effects (e.g., hemodynamics, seizures, level of consciousness) that may have influenced both medication selection and patient survival [39]. The differences could also be attributed to length–time bias: patients who died or left the ICU within 7 days did not contribute data for the full study period. Survivors were more likely to have longer ICU stays and therefore receive a greater number of continuous infusions overtime. Notably, in multivariable analysis, odds of mortality were increased with higher PRISM III scores and shorter length of stay, decreased with higher admission GCS score, and not significantly associated with center effect, age, median pediatric intensity level of therapy (PILOT) score or status epilepticus. It is conceivable that initial severity of TBI (GCS) and critical illness (PRISM III) influenced both medication selection and mortality. GCS itself may be confounded by sedative and NMB medications: in a previous analysis of the ADAPT cohort, patients with GCS scores of 3 had greater likelihood of NMB use [40]. Future prospective studies may better differentiate medication effects from these potential confounders. Fifth, bolus administrations of analgesic, sedative, and NMB medications were excluded from this analysis. We were therefore unable to comment on the trends of bolus medications given for pain, synchrony with mechanical ventilation, procedural sedation, or during periods of elevated ICP.

Finally, the ADAPT dataset was collected from 2014 to 2017. Although management practices may have changed slightly since the publication of the 2019 updated Brain Trauma Foundation guidelines, the only new recommendation contained in the 2019 guidelines concerns bolus doses, which are not analyzed in this study. Given these limitations, our findings leave more questions than answers regarding optimal analgesia, sedation, and NMB strategies in pediatric sTBI. However, this study does provide a potentially useful description of current practice. Taken together, these findings are hypothesis generating and could inform future investigations.

## Conclusions

Opioids and benzodiazepines, predominately fentanyl and midazolam, were administered to most children with sTBI in the ADAPT dataset. Although less frequent, propofol and dexmedetomidine were also used. The use of opioid infusions, specifically morphine and dexmedetomidine, were associated with survival; however, we are unable to make any recommendation about the safest or most effective analgesia, sedation, and/or NMB medication strategy for children with sTBI.

## Supplementary Information

Below is the link to the electronic supplementary material.

Supplementary file1 (DOCX 16 KB)

## References

[1] Fink EL, Kochanek PM, Tasker RC, et al. International survey of critically ill children with acute neurologic insults: the prevalence of acute critical neurological disease in children: a global epidemiological assessment study*. Pediatr Crit Care Med. 2017;18(4):330. 10.1097/PCC.0000000000001093. 28207570 10.1097/PCC.0000000000001093 PMC5380574

[2] Centers for Disease Control and Prevention. National Center for Health Statistics: Mortality Data on CDC WONDER. Accessed August 15, 2024. https://wonder.cdc.gov/Deaths-by-Underlying-Cause.html

[3] Cunningham RM, Walton MA, Carter PM. The major causes of death in children and adolescents in the United States. N Engl J Med. 2018;379(25):2468–75. 10.1056/NEJMsr1804754. 30575483 10.1056/NEJMsr1804754 PMC6637963

[4] TBI Data and Statistics|Concussion|Traumatic Brain Injury|CDC Injury Center. Accessed February 28, 2021. https://www.cdc.gov/traumaticbraininjury/data/

[5] Dewan MC, Mummareddy N, Wellons JC, Bonfield CM. Epidemiology of global pediatric traumatic brain injury: qualitative review. World Neurosurg. 2016;91:497-509.e1. 10.1016/j.wneu.2016.03.045. 27018009 10.1016/j.wneu.2016.03.045

[6] Kochanek PM, Clark RS, Ruppel RA, Adelson PD, Bell MJ, Whalen MJ, Robertson CL, Satchell MA, Seidberg NA, Marion DW, Jenkins LW. Biochemical, cellular, and molecular mechanisms in the evolution of secondary damage after severe traumatic brain injury in infants and children: lessons learned from the bedside. Pediatr Crit Care Med. 2000;1(1):4–19. 12813280 10.1097/00130478-200007000-00003

[7] Kochanek PM, Tasker RC, Carney N, et al. Guidelines for the management of pediatric severe traumatic brain injury, third edition: update of the brain trauma foundation guidelines. Pediatr Crit Care Med. 2019;20(3):S1-82. 10.1097/PCC.0000000000001735. 30829890 10.1097/PCC.0000000000001735

[8] Kochanek PM, Tasker RC, Bell MJ, et al. Management of pediatric severe traumatic brain injury: 2019 consensus and guidelines-based algorithm for first and second tier therapies. Pediatr Crit Care Med. 2019;20(3):269–79. 10.1097/PCC.0000000000001737. 30830015 10.1097/PCC.0000000000001737

[9] Shein SL, Ferguson NM, Kochanek PM, et al. Effectiveness of pharmacological therapies for intracranial hypertension in children with severe traumatic brain injury-results from an automated data collection system time-synched to drug administration. Pediatr Crit Care Med. 2016;17(3):236–45. 10.1097/PCC.0000000000000610. 26673840 10.1097/PCC.0000000000000610 PMC4779724

[10] Welch TP, Wallendorf MJ, Kharasch ED, Leonard JR, Doctor A, Pineda JA. Fentanyl and midazolam are ineffective in reducing episodic intracranial hypertension in severe pediatric traumatic brain injury. Crit Care Med. 2016;44(4):809–18. 10.1097/CCM.000000000000155. 26757162 10.1097/CCM.0000000000001558 PMC5005007

[11] Liu SY, Kelly-Hedrick M, Temkin N, et al. Association of early dexmedetomidine utilization with clinical and functional outcomes following moderate-severe traumatic brain injury: a transforming clinical research and knowledge in traumatic brain injury study*. Crit Care Med. 2024;52(4):607–17. 10.1097/CCM.0000000000006106. 37966330 10.1097/CCM.0000000000006106 PMC10939970

[12] Carney N, Totten AM, Ullman JS, et al. Guidelines for the Management of Severe Traumatic Brain Injury 4th Edition. Published online 2016

[13] Ferguson NM, Rebsamen S, Field AS, et al. Magnetic resonance imaging findings in infants with severe traumatic brain injury and associations with abusive head trauma. Children. 2022;9(7): 1092. 10.3390/children9071092. 35884076 10.3390/children9071092 PMC9322188

[14] McNamara CR, Kalinowski A, Horvat CM, et al. New functional impairment after hospital discharge by traumatic brain injury mechanism in under 3-years-old admitted to the PICU in a single center retrospective study. Pediatr Crit Care Med J Soc Crit Care Med World Fed Pediatr Intensive Crit Care Soc. 2024;25(3):250–8. 10.1097/PCC.0000000000003417. 10.1097/PCC.0000000000003417 PMC10932819 38088760

[15] Chin KH, Bell MJ, Wisniewski SR, et al. Effect of Administration of neuromuscular blocking agents in children with severe traumatic brain injury on acute complication rates and outcomes: a secondary analysis from a randomized, controlled trial of therapeutic hypothermia. Pediatr Crit Care Med J Soc Crit Care Med World Fed Pediatr Intensive Crit Care Soc. 2015;16(4):352–8. 10.1097/PCC.0000000000000344. 10.1097/PCC.0000000000000344 PMC4424136 25599147

[16] Bedell EA, DeWitt DS, Prough DS. Fentanyl infusion preserves cerebral blood flow during decreased arterial blood pressure after traumatic brain injury in cats. J Neurotrauma. 1998;15(11):985–92. 10.1089/neu.1998.15.985. 9840771 10.1089/neu.1998.15.985

[17] Rosow CE, Moss J, Philbin DM, Savarese JJ. Histamine release during morphine and fentanyl anesthesia. Anesthesiology. 1982;56(2):93–6. 10.1097/00000542-198202000-00003. 6172999 10.1097/00000542-198202000-00003

[18] Guardenier A, Peterson B, Hilfiker M, Shellington D. 1170: acute renal failure in pediatric patients after moderate to severe traumatic brain injury. Crit Care Med. 2015;43(12):294. 10.1097/01.ccm.0000475001.16167.bc.

[19] Nathan S, Sethna C, Schleien C, Schneider J. 1303: Cerebral-renal relations in pediatric traumatic brain injury. Crit Care Med. 2021;49(1):657. 10.1097/01.ccm.0000731100.40267.83.

[20] Fleischer JE, Milde JH, Moyer TP, Michenfelder JD. Cerebral effects of high-dose midazolam and subsequent reversal with Ro 15–1788 in dogs. Anesthesiology. 1988;68(2):234–42. 10.1097/00000542-198802000-00010. 3124673 10.1097/00000542-198802000-00010

[21] Flower O, Hellings S. Sedation in traumatic brain injury. Emerg Med Int. 2012;2012: 637171. 10.1155/2012/637171. 23050154 10.1155/2012/637171 PMC3461283

[22] Rahimi S, Dadfar B, Tavakolian G, Asadi Rad A, Rashid Shabkahi A, Siahposht-Khachaki A. Morphine attenuates neuroinflammation and blood-brain barrier disruption following traumatic brain injury through the opioidergic system. Brain Res Bull. 2021;176:103–11. 10.1016/j.brainresbull.2021.08.010. 34464684 10.1016/j.brainresbull.2021.08.010

[23] Kelly DF, Goodale DB, Williams J, et al. Propofol in the treatment of moderate and severe head injury: a randomized, prospective double-blinded pilot trial. J Neurosurg. 1999;90(6):1042–52. 10.3171/jns.1999.90.6.1042. 10350250 10.3171/jns.1999.90.6.1042

[24] Chiu WT, Lin TJ, Lin JW, Huang SJ, Chang CK, Chen HY. Multicenter evaluation of propofol for head-injured patients in Taiwan. Surg Neurol. 2006;66(Suppl 2):S37-42. 10.1016/j.surneu.2006.08.028. 17071254 10.1016/j.surneu.2006.08.028

[25] Kam PCA, Cardone D. Propofol infusion syndrome. Anaesthesia. 2007;62(7):690–701. 10.1111/j.1365-2044.2007.05055.x. 17567345 10.1111/j.1365-2044.2007.05055.x

[26] Kang TM. Propofol infusion syndrome in critically ill patients. Ann Pharmacother. 2002;36(9):1453–6. 10.1345/aph.1A321. 12196066 10.1345/aph.1A321

[27] Felmet K, Nguyen T, Clark RS, Orr D, Carcillo J. The FDA warning against prolonged sedation with propofol in children remains warranted. Pediatrics. 2003;112(4):1002–3. 10.1542/peds.112.4.1002. 14523206 10.1542/peds.112.4.1002

[28] Smith HAB, Besunder JB, Betters KA, et al. 2022 Society of critical care medicine clinical practice guidelines on prevention and management of pain, agitation, neuromuscular blockade, and delirium in critically Ill pediatric patients with consideration of the icu environment and early mobility. Pediatr Crit Care Med. 2022;23(2): e74. 10.1097/PCC.0000000000002873. 35119438 10.1097/PCC.0000000000002873

[29] Pandharipande PP, Pun BT, Herr DL, et al. Effect of sedation with dexmedetomidine vs lorazepam on acute brain dysfunction in mechanically ventilated patients: the MENDS randomized controlled trial. JAMA. 2007;298(22):2644–53. 10.1001/jama.298.22.2644. 18073360 10.1001/jama.298.22.2644

[30] Xu J, Xiao Q. Assessment of the effects of dexmedetomidine on outcomes of traumatic brain injury using propensity score analysis. BMC Anesthesiol. 2022;22(1):280. 10.1186/s12871-022-01822-2. 36056318 10.1186/s12871-022-01822-2 PMC9438148

[31] Kirk KA, Shoykhet M, Jeong JH, et al. Dysautonomia after pediatric brain injury. Dev Med Child Neurol. 2012;54(8):759–64. 10.1111/j.1469-8749.2012.04322.x. 22712762 10.1111/j.1469-8749.2012.04322.x PMC3393822

[32] Jerousek CR, Reinert JP. The role of dexmedetomidine in paroxysmal sympathetic hyperactivity: a systematic review. Ann Pharmacother. 2024. 10.1177/10600280231194708. 37608463 10.1177/10600280231194708

[33] Gibbs J. The effect of intravenous ketamine on cerebrospinal fluid pressure. Br J Anaesth. 1972;44(12):1298–302. 10.1093/BJA/44.12.1298. 4650346 10.1093/bja/44.12.1298

[34] List WF, Crumrine RS, Cascorbi HF, Weiss MH. Increased cerebrospinal fluid pressure after ketamine. Anesthesiology. 1972. 10.1097/00000542-197201000-00023. 5006995 10.1097/00000542-197201000-00023

[35] Peters AJ, Khan SA, Koike S, Rowell S, Schreiber M. Outcomes and physiologic responses associated with ketamine administration after traumatic brain injury in the United States and Canada: a retrospective analysis. J Trauma Inj. 2023;36(4):354–61. 10.20408/jti.2023.0034. 39381569 10.20408/jti.2023.0034 PMC11309261

[36] Bar-Joseph G, Guilburd Y, Tamir A, Guilburd JN. Effectiveness of ketamine in decreasing intracranial pressure in children with intracranial hypertension: clinical article. J Neurosurg Pediatr. 2009;4(1):40–6. 10.3171/2009.1.PEDS08319. 19569909 10.3171/2009.1.PEDS08319

[37] Laws JC, Vance EH, Betters KA, et al. Acute effects of ketamine on intracranial pressure in children with severe traumatic brain injury*. Crit Care Med. 2023;51(5):563–72. 10.1097/CCM.0000000000005806. 36825892 10.1097/CCM.0000000000005806 PMC11441348

[38] Jacobwitz M, Mulvihill C, Kaufman MC, et al. A comparison of ketamine and midazolam as first-line anesthetic infusions for pediatric status epilepticus. Neurocrit Care. 2024;40(3):984–95. 10.1007/s12028-023-01859-2. 37783824 10.1007/s12028-023-01859-2

[39] Ahmed N, Russo L, Kuo YH. Levetiracetam or phenytoin as prophylaxis for status epilepticus: secondary analysis of the “approaches and decisions in acute pediatric traumatic brain injury trial” (ADAPT) dataset, 2014–2017. Pediatr Crit Care Med J Soc Crit Care Med World Fed Pediatr Intensive Crit Care Soc. 2024;25(8):710–9. 10.1097/PCC.0000000000003526. 10.1097/PCC.0000000000003526 38717237

[40] Murphy S, Thomas NJ, Gertz SJ, et al. Tripartite stratification of the glasgow coma scale in children with severe traumatic brain injury and mortality: an analysis from a multi-center comparative effectiveness study. J Neurotrauma. 2017;34(14):2220–9. 10.1089/neu.2016.4793. 28052716 10.1089/neu.2016.4793 PMC5510706

