# Supplementary material for: Analgesia, Sedation, and Neuromuscular Blockade in Pediatric Severe Traumatic Brain Injury: Secondary Analysis of the “Approaches and Decisions in Acute Pediatric TBI Trial” (ADAPT)
Source: Neurocrit Care. 2025 Aug 15;43(3):745–55. doi: 10.1007/s12028-025-02336-8 (PMC12459016; doi:10.1007/s12028-025-02336-8)
Supplement: Supplementary file 1 — Supplementary file1 (DOCX 16 KB) [file 12028_2025_2336_MOESM1_ESM.docx]

**Supplemental Table 1. Correlation Matrix**

|  | Age (years) | Admission GCS Score | Admission AIS Score | Admission PRISM III Score | Median PILOT Score | Status Epilepticus | Barbiturate Use | ICU LOS (days) |
| --- | --- | --- | --- | --- | --- | --- | --- | --- |
| Age (years) | 1.00 |  |  |  |  |  |  |  |
| Admission GCS Score | -0.04 | 1.00 |  |  |  |  |  |  |
| Admission AIS Score | 0.24 | -0.12 | 1.00 |  |  |  |  |  |
| Admission PRISM III Score | -0.07 | -0.28 | 0.15 | 1.00 |  |  |  |  |
| Median PILOT Score | -0.04 | -0.12 | -0.01 | 0.12 | 1.00 |  |  |  |
| Status Epilepticus | -0.19 | -0.07 | -0.01 | 0.02 | 0.09 | 1.00 |  |  |
| Barbiturate Use | -0.09 | -0.12 | -0.04 | 0.07 | 0.30 | 0.11 | 1.00 |  |
| ICU LOS (days) | 0.03 | -0.12 | 0.21 | 0.11 | 0.19 | 0.12 | 0.22 | 1.00 |
